# Retraction: Tracking COVID-19 vaccine hesitancy and logistical challenges: A machine learning approach

**DOI:** 10.1371/journal.pone.0255347

**Published:** 2021-07-22

**Authors:** Shantanu Dutta, Ashok Kumar, Moumita Dutta, Caolan Walsh

After this article [1] was published, concerns came to light regarding data use permissions. The authors obtained news articles for this study on Factiva. While the authors represented to PLOS that they had legitimate permissions to access the articles, concerns were noted post-publication that the authors’ data mining of news articles on Factiva did not comply with the terms of the University of Ottawa’s license with Factiva. Therefore, the authors retract this article.

The authors requested that PLOS remove the article from online publication, and informed PLOS that the University of Ottawa library and representatives of Factiva (Dow Jones) had both requested this action. Following an internal assessment of this case, PLOS agreed to remove the article from the *PLOS ONE* website at the time of retraction.

All authors agreed with retraction.
